# Differential cell death response to photodynamic therapy is dependent on dose and cell type

**DOI:** 10.1054/bjoc.2001.1795

**Published:** 2001-05

**Authors:** L Wyld, M W R Reed, N J Brown

**Affiliations:** Section of Surgical and Anaesthetic Sciences, Division of Clinical Sciences, University of Sheffield, Floor K, Royal Hallamshire Hospital, Sheffield, S10 2JF, UK

## Abstract

PDT-induced cell death, by either apoptosis or necrosis may vary with cell type or PDT dose. 5 cell types were treated with varying doses of aminolaevulinic acid-induced PDT and the type of cell death analysed. The mode of cell death was found to depend on both cell type and light dose. © 2001 Cancer Research Campaign www.bjcancer.com


British Journal of Cancer (2001) **84**(10), 1384–1386
© 2001 Cancer Research Campaign
doi: 10.1054/ bjoc.2001.1795, available online at http://www.idealibrary.com on IDEAL®

http://www.bjcancer.com

### Differential cell death response to photodynamic therapy is dependent on dose and cell type

**L Wyld, MWR Reed and NJ Brown**

Section of Surgical and Anaesthetic Sciences, Division of Clinical Sciences, University of Sheffield, Floor K, Royal Hallamshire Hospital, Sheffield, S10 2JF, UK

**Summary** PDT-induced cell death, by either apoptosis or necrosis may vary with cell type or PDT dose. 5 cell types were treated with varying doses of aminolaevulinic acid-induced PDT and the type of cell death analysed. The mode of cell death was found to depend on both cell type and light dose. © 2001 Cancer Research Campaign http://www.bjcancer.com

Photodynamic therapy (PDT) may result in either apoptotic or necrotic cell death (Noodt et al, 1999). Many stimuli may trigger apoptosis: DNA damage, tumour necrosis factor, radiation, reactive oxygen species and elevated intracellular calcium levels. These stimuli interact with mitochondria, which are thought to be key regulators of apoptosis (Susin et al, 1998), by activation of the caspase cascade. Photodynamic therapy may interact with many of these pathways but it is unclear which is responsible for triggering apoptosis following PDT. The type of response may vary according to the cell type, the physical properties and intracellular localization of the sensitizer (Noodt et al, 1999) and the PDT dose (Kessel et al, 1995). The effect of PDT dose may reflect the fact that at low doses the cellular machinery for apoptosis is activated whereas at higher doses, the apoptotic machinery is itself damaged. The effect of cell type may depend on the genetics of the cell, as neoplastic cells often have mutations affecting the apoptotic machinery.

### MATERIALS AND METHODS

### Cell lines

**MCF 7**, Human Mammary Carcinoma (ECACC, European collection of Animal Cell Cultures, Porton Down, UK). This cell line has a wild-type p53 gene (Sharma and Srikant, 1998), but a mutation in the caspase 3 gene (Janicke et al, 1998).

**T47D**, Human Mammary Carcinoma (ECACC). This cell line contains a mutated p53 gene (Bonsing et al, 1997).

**HT1197**, Human Bladder Carcinoma (ECACC). This cell line contains a mutated p53 gene (Cooper et al, 1994).

**WRC**, Walker Rat Carcinoma. This cell line contains a wild-type p53 gene (Tang et al, 1996).

**MVECs**, Human Microvascular Endothelial Cells, derived from human adipose tissue, by the method of Hewitt and Murray (1993).

*Received 5 April 2000*
*Revised 30 January 2001*
*Accepted 12 February 2001*

*Correspondence to:* NJ Brown

### Cell culture techniques

All of the cell types studied were cultured in their optimal media and maintained using standard tissue culture techniques.

### Cell death assay: dual staining for apoptosis and necrosis

The assay is based on the concurrent use of 2 fluorescent DNA stains, propidium iodide and Hoechst 33342. Propidium iodide (PI) stains the nuclei of necrotic cells red (Crissman, 1995). Hoechst 33342 stains viable and apoptotic nuclei green (Crissman, 1995). Apoptotic and viable cells are discriminated by nuclear morphology. Cells were plated into 96-well plates and treated with ALA-PDT at light doses previously calculated to kill 50 or 90% of cells ($LD_{50}$ or $LD_{90}$ light dose). 1 mM ALA was added to the cells for 4 hours and the cells exposed to violet light (350 to 450 nm, 86.5 $mW.cm^{-2}$), at light doses varying between 0.5 $J.cm^{-2}$ and 25 $J.cm^{-2}$. Controls for light alone, ALA alone and no light/no ALA were also studied. After 30 minutes, 2, 8 and 24 hours, the cells were assayed for viability and type of death response by staining with PI and Hoechst 33342. The total number of necrotic, viable and apoptotic nuclei were counted per high power field per well. 9 repeats were performed.

The presence of apoptotic cells was confirmed by TUNEL labelling (Terminal deoxynucloetidyl transferase-mediated deoxy Uridine triphophate Nick End Labelling; Gavrieli et al, 1992). Following PDT, the cells were fixed in paraformaldehyde, permeabilized in ethanol and treated with the terminal deoxynucleotidyl transferase enzyme (TdT), which catalyses the binding of fluorescent deoxyuridine triphosphate (fluorescein isothiocyanate, FITC-dUTP), to the ends of the DNA strand breaks. The fluorescein label was then detected by fluorescence microscopy.

### RESULTS

The light doses required to cause 50 and 90% cell death respectively varied with cell line: HT1197: 0.5 and 3, MCF-7: 4 and 8, T47D: 1.5 and 4, WRC: 2 and 5 $J.cm^{-2}$. For MVEC an $LD_{50}$ light dose only was studied, and was found to be 25 $J.cm^{-2}$. At the $LD_{90}$ light dose for MVECs, some toxicity occurred in the light only control and therefore this light dose was not studied further. In all other experiments, no significant toxicity was noted with either light alone or ALA alone.

**Table 1** Table showing type of cell death response after $LD_{50}$ and $LD_{90}$ photodynamic therapy for a range of cell types

| Time after PDT | Percentage of cell death by cell type and PDT dose intensity. | | | | | | | | |
|---|---|---|---|---|---|---|---|---|---|
| | HT1197 $LD_{50}$ | HT1197 $LD_{90}$ | MCF-7 $LD_{50}$ | MCF-7 $LD_{90}$ | T47D $LD_{50}$ | T47D $LD_{90}$ | WRC $LD_{50}$ | WRC $LD_{90}$ | MVEC $LD_{50}$ |
| Necrosis | | | | | | | | | |
| 0.5 hours | 6.7 [1.8] | 77.7 [7.2] | 12.7 [2.17] | 53.2 [7.6] | 24.9 [8.3] | 75.9 [6.6] | 8 [2.4] | 30.4 [8.06] | 0.9 [0.5] |
| 2 hours | 10.9 [3.0] | 64.3 [11.4] | 18.9 [4.7] | 61.1 [5.9] | 51.1 [9.7] | 70.1 [8.9] | 17.6 [3.9] | 75.8 [7.2] | 3.8 [1.2] |
| 8 hours | 7.5 [2.4] | 52.9 [12.0] | 25 [4.4] | 82.1 [7.4] | 57.5 [9.4] | 86.7 [8.2] | 27.3 [6.9] | 64.7 [11.5] | 5.7 [1.2] |
| 24 hours | 14.5 [7.9] | 73.4 [7.5] | 31 [4.8] | 87.5 [5.4] | 51.4 [6.3] | 75.7 [12.1] | 43.3 [6.0] | 87.5 [5.09] | 11.2 [1.2] |
| Apoptosis | | | | | | | | | |
| 0.5 hours | 8.7 [2.8] | 1.1 [0.5] | 0 | 0 | 0 | 0 | 0 | 0 | 1.6 [0.27] |
| 2 hours | 27.3 [8.2] | 28.4 [10.5] | 0 | 0 | 0 | 0 | 0 | 0 | 5.1 [0.8] |
| 8 hours | 55 [6.6] | 25.8 [8.3] | 0 | 0 | 0 | 0 | 0.5 [0.2] | 0 | 11.1 [1.13] |
| 24 hours | 10.5 [3.5] | 4.4 [1.8] | 0 | 0 | 0 | 0 | 3.0 [0.37] | 0 | 30.1 [2.2] |

Data shown are means plus standard errors [ ]. Necrotic cells were identified by nuclear staining with propidium iodide with normal nuclear morphology. Viable cells and apoptotic cells were identified by staining with Hoechst 33342, and differentiated by nuclear morphology.

### HT 1197 cells, $LD_{50}$ and $LD_{90}$ doses

Following a $LD_{50}$ light dose, this cell line died predominantly by apoptosis, occurring maximally 8 hours after treatment, but apparent by 30 minutes. Following an $LD_{90}$ light dose, cell death was predominantly by necrosis (Table 1).

### MCF-7, $LD_{50}$ and $LD_{90}$ doses

Following both a $LD_{50}$ and a $LD_{90}$ light dose, necrotic cell death only was apparent by 30 minutes and increased to a peak at 24 hours (Table 1).

### T47D, $LD_{50}$ and $LD_{90}$ doses

Following a $LD_{50}$ and a $LD_{90}$ light dose necrotic cell death was apparent by 30 minutes and increased progressively over the study period to a peak at 8 hours, followed by a slight decrease at 24 hours (Table 1).

### WRC, $LD_{50}$ and $LD_{90}$ doses

This cell line responded to $LD_{50}$ PDT predominantly by necrosis but at 24 hours apoptotic cells appeared.

Following an $LD_{90}$ dose this cell line responded to PDT predominantly by necrosis. Unlike at the $LD_{50}$ dose, no significant apoptosis was noted (Table 1).

### MVECs, $LD_{50}$ dose

MVECs responded to PDT predominantly by apoptosis, of slower onset than with HT1197 cells (Table 1).

### TUNEL staining

Following PDT, only HT1197 cells, MVECs and WRC cells demonstrated apoptosis. This was confirmed by TUNEL staining. MCF-7 and T47D cells did not stain.

### DISCUSSION

Photodynamic therapy causes cell death by apoptosis and necrosis, depending on the type of cell and the PDT dose.

The time course of the apoptotic response varies between cell lines, suggesting that more than one apoptotic pathway may be involved. The p53 gene is probably not a key mediator of PDT-induced apoptosis as a p53 mutant cell line (HT1197), died predominantly by apoptosis.

The PDT light dose dependency observed in this study for HT1197 and WRC cells concurs with other studies, demonstrating apoptosis at low doses which is overwhelmed at higher doses (Kessel et al, 1995).

The MCF-7 cell line did not respond to $LD_{50/90}$ PDT by apoptosis dying by necrosis only possibly because these cells contain a mutated form of caspase 3 (Janicke et al, 1998) an important downstream effector of apoptosis.

Mutations in the genes controlling apoptosis, such as *bcl-2* and *p53*, are common in malignancies. There is limited research investigating the effect of such mutations on PDT-sensitivity. Since a number of pathways exist whereby PDT can trigger apoptosis, this suggests that complete resistance to PDT would be uncommon. Even in cells where there is failure of one of the steps in the final common pathway for apoptosis, namely one of the downstream caspases (such as is the case with the MCF-7 cell line; Janicke et al, 1998), the cells will respond by necrosis. This redundancy of death pathways suggests that treatment failures with PDT should be rare, provided the appropriate dose is administered.

### ACKNOWLEDGEMENTS

This work was supported by the Trustees of the Former United Sheffield Hospitals. The method for TUNEL staining was suggested by Dr M Sharrard of York University. Extraction, purification and characterisation of MVECs was performed by Mr JL Burn, Sheffield University.

*© 2001 Cancer Research Campaign* 

### REFERENCES

Bonsing BA, Corver WE, Gorsira MC, Van Vliet M, Oud PS, Cornelisse CCJ and Fleuren GJ (1997) Specificity of seven monoclonal antibodies against p53 evaluated with Western blotting, immunohistochemistry, confocal laser scanning microscopy and flow cytometry. *Cytometry* **28**: 11–24

Cooper MJ, Haluschak JJ, Johnson D, Schwartz S, Morrison LJ, Lippa M, Hatzivassiliou G and Tan J (1994) P53 mutation in bladder carcinoma cell lines. *Oncol Res* **6**: 569–579

Crissman HA (1995) Cell cycle analysis by flow cytometry. In: *Cell Growth and Apoptosis*, 2nd Edition. Studzinski GP (ed.). pp 21–43. IRL Press: Oxford

Gavrieli Y, Sherman Y and Ben-Sasson SA (1992) Identification of programmed cell death in situ via specific labelling of nuclear DNA fragmentation. *J Cell Biol* **119**: 493–501

Hewett PW and Murray JC (1993) Human microvascular endothelial cells: isolation, culture and characterisation. In Vitro *Cell Develop Biol* **12**: 823–830

Janicke RU, Sprengart ML, Wati MR and Porter AG (1998) Caspase 3 is required for DNA fragmentation and morphological changes associated with apoptosis. *J Biol Chem* **273**: 9357–9360

Kessel D, Luo Y, Woodburn K, Chang C and Henderson BW (1995) Mechanism of phototoxicity catalysed by two porphycenes. *SPIE*, **2392**: 122–128

Noodt BB, Berg K, Stokke T, Peng Q and Nesland JM (1999) Different apoptotic pathways are induced from various intracellular sites by tetraphenylporphyrins and light. *Br J Cancer* **79**: 72–81

Sharma K and Srikant CB (1998) Induction of wild type p53, Bax and acidic endonuclease during somatostatin-signalled apoptosis in MCF-7 human breast cancer cells. *Int J Cancer* **76**: 259–266

Susin SA, Zamzani N and Kroemer G (1998) Mitochondria as regulators of apoptosis: doubt no more. *Biochim Biophys Acta* **1366**: 151–165

Tang DG, Chen YQ and Honn KV (1996) Arachidonate lipoxygenases as essential regulators of cell survival and apoptosis. *Proc Nat Acad Sci* **93**: 5241–5246

 *© 2001 Cancer Research Campaign*
